# 3D Kidney Segmentation from Abdominal Images Using Spatial-Appearance Models

**DOI:** 10.1155/2017/9818506

**Published:** 2017-02-09

**Authors:** Fahmi Khalifa, Ahmed Soliman, Adel Elmaghraby, Georgy Gimel'farb, Ayman El-Baz

**Affiliations:** ^1^Bioengineering Department, University of Louisville, Louisville, KY, USA; ^2^Electronics and Communication Engineering Department, Mansoura University, Mansoura, Egypt; ^3^Computer Engineering and Computer Science Department, University of Louisville, Louisville, KY, USA; ^4^Department of Computer Science, University of Auckland, Auckland, New Zealand

## Abstract

Kidney segmentation is an essential step in developing any noninvasive computer-assisted diagnostic system for renal function assessment. This paper introduces an automated framework for 3D kidney segmentation from dynamic computed tomography (CT) images that integrates discriminative features from the current and prior CT appearances into a random forest classification approach. To account for CT images' inhomogeneities, we employ discriminate features that are extracted from a higher-order spatial model and an adaptive shape model in addition to the first-order CT appearance. To model the interactions between CT data voxels, we employed a higher-order spatial model, which adds the triple and quad clique families to the traditional pairwise clique family. The kidney shape prior model is built using a set of training CT data and is updated during segmentation using not only region labels but also voxels' appearances in neighboring spatial voxel locations. Our framework performance has been evaluated on in vivo dynamic CT data collected from 20 subjects and comprises multiple 3D scans acquired before and after contrast medium administration. Quantitative evaluation between manually and automatically segmented kidney contours using Dice similarity, percentage volume differences, and 95th-percentile bidirectional Hausdorff distances confirms the high accuracy of our approach.

## 1. Introduction

**Figure 1:**
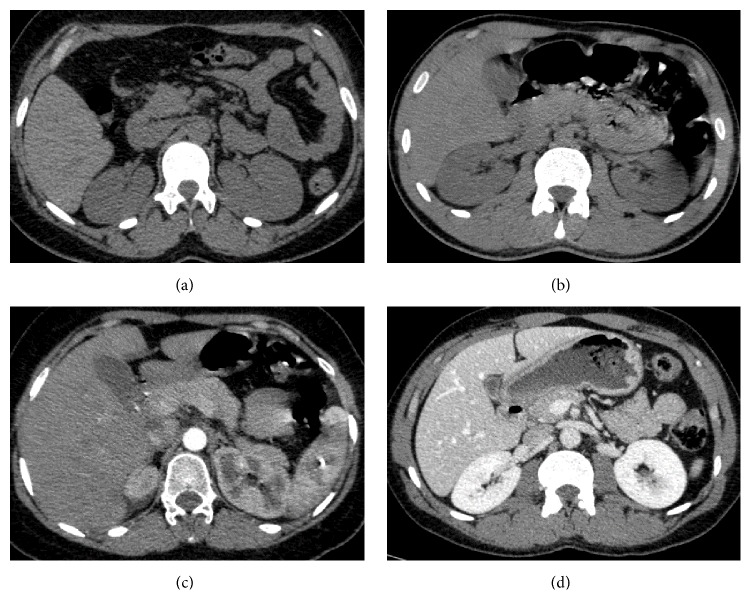
Axial cross-sectional images showing different CT data challenges: (a) low contrast, (b) intensity inhomogeneities, (c) fuzzy boundary, and (d) contrast and anatomy differences.

Kidney segmentation from dynamic contrast-enhanced computed tomography (CT) is of immense importance for any computer-assisted diagnosis of renal function assessment, pathological tissue localization, radiotherapy planning, and so forth [1]. Nevertheless, accurate segmentation of kidney tissues from dynamic CT images is challenging due to many reasons, including data acquisition artifacts, large inhomogeneity of the kidney (e.g., cortex and medulla), large anatomical differences between subjects, similar intensities of adjacent organs, and varying signal intensities over the time course of data collection due to agent transit [2, 3]; see Figure 1.

Many automated and semiautomated approaches have been developed to address these challenges. Earlier computerized renal image analysis (e.g., [4]) was usually carried out either manually or semiautomatically. Typically, a user-defined region-of-interest (ROI) is delineated in one image and for the rest of the images, image edges were detected and the model curve was matched to these edges. However, ROI placements are based on the users' knowledge of anatomy and thus are subject to inter- and intraobserver variability. Additionally, these methods are very slow, even though semiautomated techniques reduce the processing time. Traditional segmentation techniques utilizing image thresholding or region growing [5–9] have been also explored for CT kidney segmentation. For example, Pohle and Toennies [7] developed an automatic region-growing algorithm based on estimating the homogeneity criterion from the characteristics of the input images. A semiautomated method was also proposed by Leonardi et al. [9]. First, a region-growing approach is performed to obtain an initial kidney segmentation from the grayscale image stack. Then, a refinement step utilizing histogram analysis is employed to the initially segmented kidney regions to reduce incorrectly segmented areas. However, these traditional methods are not accurate due to the large overlap of gray level intensity between the kidney and other surrounding tissues in addition to sensitive to initialization.

To more accurately segment abdominal CTs, recent segmentation methods consider either visual appearances, prior shapes, texture features, or hybrid techniques. In particular, Tsagaan et al. [10] presented a deformable model-based approach utilizing a nonuniform rational B-spline surface representation. Their framework incorporated statistical shape information (e.g., mean and variation) into the objective function for the model fitting process as an additional energy term.

A constrained optimization deformable contour by Wang et al. [11] exploited the degree of contour interior homogeneity as an extra constraint within the level set energy minimization framework. Lu et al. [12] developed a coarse-to-fine approach for kidney segmentation on abdominal CT images using the Chan-Vese (CV) level set method [13]. Mathematical morphology operations are performed to extract the kidney structures interactively with prior anatomy knowledge. Huang et al. [14] proposed a multiphase level set approach with multidynamic shape models to segment the kidneys on abdominal CT images. Campadelli et al. [15] proposed an automatic, gray-level based segmentation framework based on a multiplanar fast marching method. A stochastic level set-based framework by Khalifa et al. [16, 17] integrated probabilistic kidney shapes and image signals priors into Markov random field (MRF) for abdominal 3D CT kidney segmentation. Despite their popularity, deformable model-based methods fail in the case of excessive image noise, poor image resolution, or diffused boundaries if they do not take advantage of a priori models.

Freiman et al. [18] proposed a model-based framework utilizing maximum a posteriori-MRF (MAP-MRF) estimation of the input CT image. The MAP-MRF estimation is obtained by using a graph min–cut technique. Lin et al. [19] proposed a framework that combined region- and model-based methods. Initial kidney location is estimated using geometrical location, statistical information, and a priori anatomical knowledge. Secondly, an elliptic candidate kidney region extraction approach is proposed. Finally, an adaptive region-growing approach is employed for kidney segmentation. Spiegel et al. [20] proposed an active shape model (ASM) based framework that was combined with a curvature-based nonrigid registration approach to solve the point correspondence problem of the training data. A hybrid framework by Chen et al. [21] combined active appearance model (AAM), live wire, and graph-cuts methods for 3D abdominal organ segmentation. In general, parametric shape-based techniques depend on the existence of adequate texture features in abdominal images and may perform poorly due to noise and the lack of well-defined features. Cuingnet et al. [22] exploited random regression and classification forests for CT kidney images segmentation. Initially, global contextual information is used to detect the kidney. This is followed by a cascade of local regression forests for refinement. Then, probabilistic segmentation maps are built using classification forest. Finally, an implicit template deformation algorithm driven by these maps is employed to obtain the final segmentation. A model-based framework by Badakhshannoory and Saeedi [23] combined low-level segmentation schemes with a statistical-based modeling approach. First, an organ space is built using a statistical model and principle component analysis. Then, each image slice of an input CT volume is segmented multiple times using a graph-based segmentation by varying segmentation parameters. Finally, a distance-based criterion from the organ space is used to choose the closest candidate as the best segmentation result. In general, knowledge-based approaches are computationally intensive, and their accuracy depends on the training data size.

Bagci et al. [24] developed a multiobject segmentation framework that integrates a statistical shape model and hierarchical object recognition into a global graph-cuts segmentation model. Wolz et al. [25] developed a hierarchical two-step atlas registration framework for multiobject segmentation. First, subject-specific priors are generated from an atlas database based on multiatlas registration and patch-based segmentation. Final segmentation is obtained using graph-cuts, incorporating high-level spatial knowledge and a learned intensity model. Another study by Okada et al. [26] performed multiobject segmentation using probabilistic atlases that combines interorgan spatial and intensity a priori models. Despite the potential to improve the segmentation accuracy due to the spatial kidney constraints from other organs, multiobject segmentation schemes require more comprehensive prior information. A semiautomated GrowCut algorithm by Dai et al. [27] employed a monotonically decreasing function and image gray features to propagate initial user-defined labels over all the slices to derive an optimal cut for a given CT data in space. Zhao et al. [28] proposed a sliced-based framework for 3D kidney segmentation. First, an initial segmentation is obtained using the CV approach [13]. Then, a set of contextual features (e.g., slices overlap, the distance) and multiple morphological operations are used to estimate the continuity between slices. The final segmentation is obtained by discarding the leakage and the weak edges between adjacent slices using a local iterative thresholding method. Chu et al. [29] presented an automated MAP-based multiorgan segmentation method that incorporated image-space division and multiscale weighting scheme. Their framework is based on a spatially divided probabilistic atlases and the segmentation is performed using a graph cut method. Yang et al. [30] developed on multiatlas framework using a two-step approach to obtain coarse-to-fine kidney segmentation. A coarse segmentation is obtained by registering an input down-sampled CT volume with a set of low-resolution atlas images. Then, cropped kidney images are coaligned with high-resolution atlas images using B-Splines registration. The final segmentation result is obtained by majority voting of all deformed labels of all atlas images. Liu et al. [31, 32] developed a framework for kidney segmentation on noncontrast CT images using efficient belief propagation. A preprocessing step is applied to extract anatomical landmarks to localize kidney search regions. Then, an efficient belief propagation is used to extract the kidney by minimizing an energy function that incorporates intensity and prior shape information. However, the method was evaluated on five noncontrast CT data sets only and additional segmentation of other organs (e.g., liver, spleen) is required to determine subimages that envelope the kidneys.

In summary, during the last few years there have been numerous studies for abdominal CTs kidney segmentation. In addition to the above-mentioned limitations, current methods have the following shortcomings. Most of them are based on visual appearance and did not take into account the spatial interaction relationships. Most of the shape-based methods utilize fixed models and therefore have limited accuracy for CT data outside their training scope. Most of the existing methods work very well with contrast CTs only. Most of the energy-based methods (e.g., graph-cut) use regional and boundary information that may not exist in some (e.g., precontrast) images and may not achieve globally optimal results.

To account for these limitations, we developed a 3D kidney segmentation framework that integrates, in addition to the current CT appearance features, higher-order appearance models and adaptive shape model features into a random forests (RF) classification model [33]. The integrated features increase the ability of our framework to account for the large CT images' inhomogeneities and therefore accurately segment both contrast and noncontrast CTs. Particularly, the spatial features are based on a higher-order Markov–Gibbs random field (MGRF) model that adds to the traditional pairwise cliques [34] the families of the triple and quad cliques. The spatial-appearance kidney shape prior is an adaptive model that is updated during segmentation and accounts not only for region labels, but also intensities in neighboring spatial locations. Moreover, compared to other tissue classification methods the RF is employed due to its (i) powerful ability to learn the characteristics of complex data classes [35], (ii) less sensitivity to data outliers, (iii) ability to overcomes overfitting of the training set, and (iv) ability to handle high dimensional spaces as well as large number of training examples.

A detailed description of our developed methodology for kidney segmentation from dynamic CT images including the details of the discriminative features is given in Section 2. It is worth mentioning that, in addition to our methodology presentation in [33], this paper provides (i) a more comprehensive review of the related literature work on the abdominal CT images segmentation (Section 3); (ii) detailed description of the metrics that are used for segmentation evaluation of our and compared techniques (Section 3); and (iii) expansion of the experimental results by adding an essential metric that is used to evaluate the robustness of segmentation techniques, namely, the receiver operating characteristics (ROC) (Section 4).

## 2. Methods

**Figure 2:**
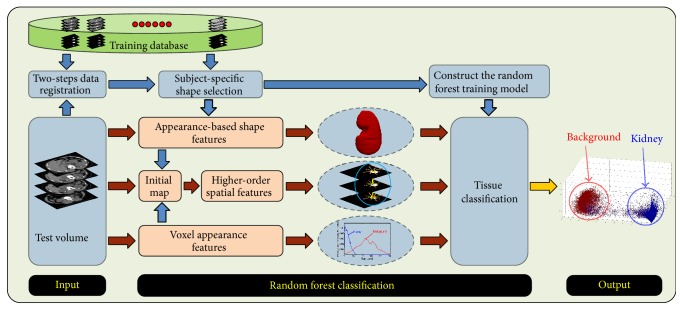
Block diagram of our kidney segmentation framework from abdominal CT images using random forest (RF).

A block diagram of our kidney segmentation framework is shown in Figure 2. Our technique is based on random forests (RF) classification and incorporates spatial-appearance features for better separation of the CT data classes. RF is an efficient multiclass machine learning technique, which is increasingly being utilized in data clustering as well as image classification. As an ensemble learning classifier, RF typically consists of many decision trees (DTs) and combines two main concepts [36]. The first is the random selection of features and the second is “bagging” [37], which implies the training of each DT with a randomly chosen and overlapping subset of the training samples. In general, as numbers of the DTs increase the results get better. Nevertheless, there is a threshold beyond which the performance benefit from adding more DTs will be lower than the computational cost for learning these additional DTs [38].

**Figure 3:**
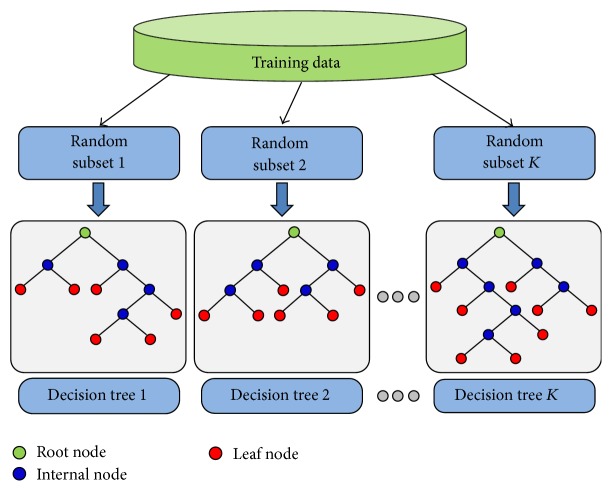
A schematic illustration of the random decision trees for random forests (RF) classification.

During the RF training phase, each DT recursively processes its randomly selected training samples' features along a path starting from the tree's root node using binary classification tests, as shown in Figure 3. The latter tests compare the features' values at each internal tree node to a certain threshold that is selected using a certain criterion. A leaf node of the DT is reached if all samples belong to a single class; the number of data samples is smaller than a predefined value, or the maximum tree depth is reached [35]. Once occurred, the most frequent class label of the training data at the node is stored for the testing phase. For testing, a given data sample is handled by applying respective tests in line with the path it traverses from the tree root node to the leaf. When a leaf node is reached, the DT casts a vote corresponding to the class assigned to this node in the training stage. Finally, a majority voting is used to class-label test samples. The final class probabilities are estimated by the fraction of votes for that class by all DTs.

In order to build an accurate RF model that provides better separation of data classes, discriminative and robust features are needed. Therefore, in this paper multiple features from the CT data are extracted, for both training and testing phases. These features include (i) first-order appearance (Hounsfield units (Hus) values) features; (ii) higher-order spatial interaction features; and (iii) appearance-based shape model features. Those features are extracted at each voxel's location **p** = (*x*, *y*, *z*) in the 3D arithmetic lattice **R** = {(*x*, *y*, *z*)  0 ≤ *x* ≤ *X* − 1, 0 ≤ *y* ≤ *Y* − 1, 0 ≤ *z* ≤ *Z* − 1} supporting the grayscale CT images, **g** = {*g*_**p**_ : **p** ∈ **R**, *g*_**p**_ ∈ **Q**}, and their region, or segmentation maps, **m** = {*m*_**p**_ : **p** ∈ **R**, *m*_**p**_ ∈ **L**}. Here, **Q** = {0,1,…, *Q* − 1} and **L** = {“KT”, “OT”} is a finite set of integer gray levels and region labels (kidney object tissues “KT” and other background tissues “OT”), respectively. Since spatial and shape features are based on probabilistic models, the first-order appearance-based features were also normalized to reduce the domination of a specific feature during RF classification. Details of the employed features are given in the following sections.

### 2.1. First-Order Appearance Features

The first type of features that are used in our framework is the CT voxel-appearance features. Those features were extracted at each voxel** p** regionally from the CT data after normalization. Due to image noise presence and reconstruction artifacts, we used, at each voxel** p**, regional intensity features in addition to the local CT Hounsfield units (HU). Namely, we used the mean HU values of a symmetric 3D cube (i.e., voxels' 26-neighbors) centered around** p** and the mean of the HUs of a 3* ×* 3 in-plane symmetric window (i.e., voxels' 8-neighbors) centered around** p**.

### 2.2. Shape Prior Features

The ultimate goal is to accurately segment the kidney from the CT data such that the extracted kidney borders closely approximate the expert manual delineation. However, due to the similar visual appearance between some kidney structures (e.g., medulla) and background, the segmentation should not rely only on image signals. Therefore, shape features of the expected kidney shape are also used in our segmentation framework. In this paper, we employed an adaptive, probabilistic kidney shape model that takes into account not only voxels' location, but also their intensity information [39, 40].

For training, a shape database is constructed using a set of training data sets that is collected from different subjects; each contains multiple CT scans acquired at different phases of contrast-enhancements. The ground truth segmentation (labeled data) of the training images is obtained by manual delineation of the kidney borders by an expert. In order to reduce the variability across subjects and maximize overlaps of the kidneys for estimating the shape prior probability, the training grayscale images are coaligned using a two-step registration methodology. First, a 3D affine transformation is used with 12 degrees of freedom (3 for the 3D translation, 3 for the 3D rotation, 3 for the 3D scaling, and 3 for the 3D shearing) to account for global motion [41]. Second, local kidney deformations are handled using a 3D B-splines based transformation proposed in [42]. Finally, the obtained transformation parameters for each scan are applied to its binary (labeled) data to be used during segmentation to estimate the shape prior probability.

For testing, an input grayscale 3D CT kidney image, **g**_**t**_, to be segmented is first coaligned with the training database using the two-step registration methodology described above. Then, a subject-specific shape, **g**_**i**_, *i* = 1,2,…, *N*, is extracted by computing the conventional normalized cross correlations (NCC) between the coaligned input grayscale image and all grayscale images in the database, to select the top *N* similar kidneys (*N* = 19 in our experiments below). Finally, visual appearances of both the input 3D grayscale CT image and the selected grayscale training images guide adapting the shape prior. Namely, the voxel-wise probabilities, *P*_*s*:**p**_(*l*) for the adaptive shape prior *P*_*s*_(**m**) = ∏_**p**∈**R**_*P*_*s*:**p**_(*m*_*p*_), are estimated based on the found voxels *l* ∈ **L**. Let **v**_*i*:**p**_(*l*) = {*ρ* : *ρ* ∈ **R**; *ρ* ∈ **C**_**p**_; |*g*_*i*:*ρ*_ − *g*_*t*:**p**_ | ≤ *τ*} be a subset of similar training voxels within a search cube **C**_**p**_ in the training image *g*_*i*_, where *τ* is a predefined fixed signal range and *g*_*t*:*p*_ is the mapped input signal. Let *v*_*i*:**p**_ = card(**v**_*i*:**p**_) denote the cardinality (number of voxels) of this subset **v**_**p**_ = ∑_*i*=1_^*N*^**v**_*i*:**p**_ and *δ*(*z*) be the Kronecker's delta-function: *δ*(0) = 1 and 0 otherwise. Then *P*_*s*:**p**_(*l*) is given as [39]

(1) $$\begin{array}{c} P_{s:p}\left(\;l\;\right)=\frac{1}{v_{p}}\overset{N}{\underset{i=1}{\sum }}\underset{\rho \in v_{i:p}}{\sum }\delta \left(\;l-m_{i:\rho }\;\right). \end{array}$$

More details about the adaptive shape model can be found in [39, 40]. Our experiments were conducted using three shape features, like the voxel-appearance features. Namely, we used the *P*_*s*_(**m**) value at** p**, the average *P*_*s*_(**m**) value for the 26 neighbors of a 3D cube around** p**, and the average *P*_*s*_(**m**) of the 8 in-plane neighbors for a 3* ×* 3 symmetric window centered at** p**.

### 2.3. Spatial Features

To improve the segmentation accuracy and account for the large inhomogeneity of the kidney, we incorporated into our segmentation approach the spatial features that describe the relationships between the kidney voxels and their neighbors. These relationships are described using a higher-order spatial model with analytically estimated potentials. The spatial modeling enhances the segmentation by calculating the likelihood of each voxel to be kidney or background on the basis of the initial labeling,** m**, of the adjacent voxels, formed by a voxel-wise classification using shape and intensity values Our spatial interactions model adds the triple and quad clique families to the traditional pairwise clique family [34] using the 18-connectivity neighborhood. Thus, it is an extension of the conventional Potts model [43], differing only in that the potentials are estimated analytically. For more mathematical details about our higher-order spatial model, please see [33, 44]. Similar to the other features, three spatial-based features were used: the local spatial probability at** p** and the average probabilities for a 3D cube and a 3 × 3 window centered around** p**. In total, the whole segmentation approach is summarized in Algorithm 1.

**Algorithm 1 (3D kidney segmentation steps).**

 

 

*Step  1 (data coalignment and shape database selection)*

- Register the input grayscale CT volume to the training database using the two-step registration in Section 2.2.
- Calculate the NCC between the input coaligned data and all training volumes. Then, select the NCC-19-top ranked training samples.

*Step  2 (features extraction)*

- Estimate the voxel-appearance features of the coaligned CT volume.
- Estimate the higher-order Potts-MGRF spatial probabilities *P*_*G*_(**m**).
- Estimate the appearance-based shape prior *P*_*s*_(**m**) using the method described in [39, 40].

*Step  3 (RF training)*

- Construct the RF training model for the selected 19-top-ranked training images.

*Step  4 (tissue segmentation)*

- Obtain the final segmentation of the input CT volume using the model in Step  3.

## 3. Segmentation Evaluation Metrics

The performance of our segmentation is evaluated using two metrics. The first is a volumetric-based similarity that characterizes spatial overlaps and volume differences between the segmented and “ground-truth” kidney regions. This type of metrics is important for studying area measurements, for example, total kidney volumes. The second is a distance-based metric that measures how close the edge of a segmented region is to the ground truth, that is, how accurate the shape of a segmented object is with respect to ground truth. Here, we used the Dice coefficient (DC) and percentage volume difference (PVD) to describe the volumetric-based similarity, while the bidirectional 95th-percentile Hausdorff distance (BHD_95_) is used to characterize the distance-based error metric: **G**↔**S**.

**Figure 4:**
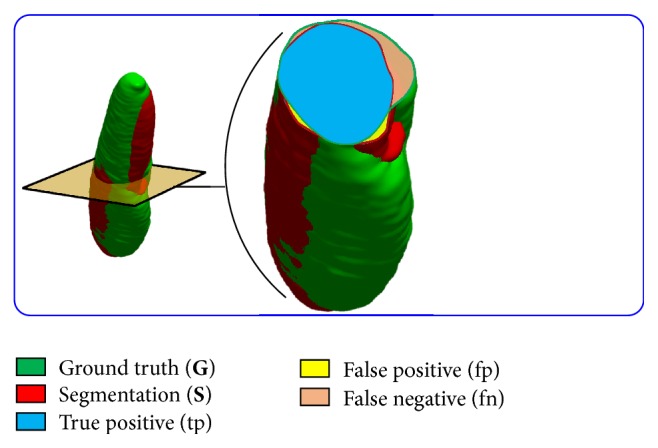
3D illustration of DC measurement for segmentation evaluation between the ground truth** G** and model segmentation** S**.

Let** G** and** S** denote sets of ground-truth and segmented kidney voxels, respectively. The similarity volumetrics evaluate an overlap between these sets and account for cardinalities (i.e., voxel numbers) *c*_*i*_ = |*V*_*i*_| of true positive (tp), false positive (fp), and false negative (fn) subsets *V*_*i*_;  *i* ∈ {tp, fp, fn}; see Figure 4.

The subsets contain true kidney voxels labeled as kidney, nonkidney (background) voxels labeled as kidney, and true kidney voxels labeled as background, respectively:

(2) Vtp=v:v∈G,v∈S;ctp=VtpVfp=v:v∉G,v∈S;cfp=VfpVfn=v:v∈G,v∉S;cfn=Vfn.

Obviously, **G** = *V*_tp_ ∪ *V*_fn_; **S** = *V*_tp_ ∪ *V*_fp_; *V*_tp_ = **G**∩**S**; and *V*_tp_ ∪ *V*_fp_ ∪ *V*_fn_ = **G** ∪ **S** where ∪ and ∩ denote the set union and intersection, respectively. Therefore, it holds that |**G** | = *c*_tp_ + *c*_fn_; |**S** | = *c*_tp_ + *c*_fp_, and |**G** ∪ **S** | = *c*_tp_ + *c*_fp_ + *c*_fn_. The DC [45] and the PVD are defined as

(3) DC=1002ctp2ctp+cfp+cfn≡1002G∩SG+SPVD=100ctp+cfn−ctp+cfpctp+cfn≡100G−SG.

**Figure 5:**
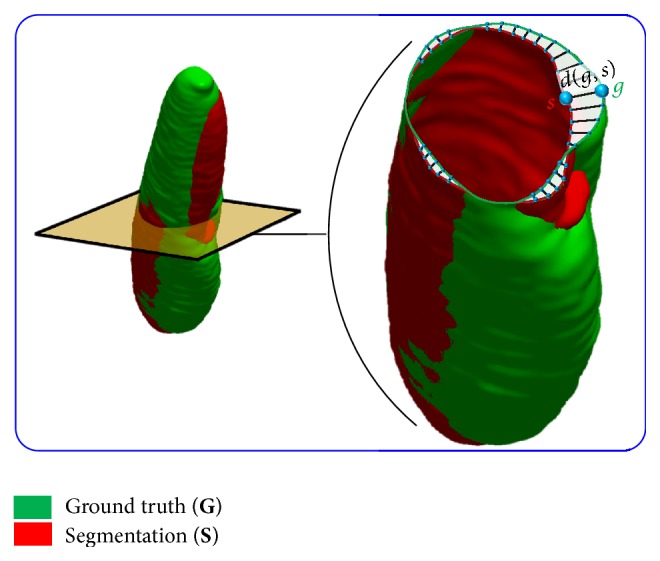
Schematic illustration for the calculation of the Haussdorf distance between the ground truth (green) and segmented (red) objects.

In addition to the DC and PVD, the 95th-percentile bidirectional Hausdorff distance (BHD_95_) is used to measure dissimilarities between the **G** and **S** boundaries; see Figure 5. The HD from** G** to** S** is the maximum distance from the points *g* from** G** to their closest points *s* in** S** [46]:

(4) $$\begin{array}{c} {HD}_{G\to S}=\max_{g\in G}\left\{\;\min_{s\in S}d\left(\;g,s\;\right)\;\right\}, \end{array}$$

where *d*(*g*, *s*) is the Cartesian distance between two 3D points. The HD is asymmetric, as generally HD_*G*→*S*_ ≠ HD_*S*→*G*_. The symmetric BHD between these two sets is defined as

(5) $$\begin{array}{c} {HD}_{G↔S}=\max\left\{\;{HD}_{G\to S},{HD}_{S\to G}\;\right\}. \end{array}$$

To decrease the sensitivity to outliers, the 95th-percentile BHD is used in this paper to measure the segmentation accuracy.

## 4. Experimental Results

Performance assessment of our framework is carried using dynamic CT kidney data, which were collected from 20 subjects. Each subject dataset consists of three 3D CT scans obtained at the pre- and postcontrast medium administration, namely, noncontrast, postcontrast, and late contrast 3D scan. The CT data were obtained using a GE light speed plus scanner (General Electric, Milwuakee, USA). The CT data acquisition parameters were 120 KV, 250 mA, in-plane resolution: 0.64 × 0.64 mm^2^, slice thickness: 0.9 mm, field-of-view (FOV): 360 mm, the 3D image sizes range from 512 × 512 × 232 to 512 × 512 × 366. In order to minimize the effect of interobserver variability, two experts delineated the kidney borders independently on the CT images and the ground truth labels were considered as the common segmented region of their delineations.

Quantitative evaluation is performed using a leave-one-subject-out approach and the number of decision trees was set to 400. First, all the 3D CT scans (60 scans in total) from all of the 20 subjects are coregistered using our registration methodology described in Section 2.2. To segment a test subject, all of its pre- and postcontrast scans are removed from the training database. Then, the 19 NCC-top-ranked scans are selected from the remaining training scans to build the test scan adaptive shape prior, described by (1) and the method in [39, 40]. Lastly, all regional features described in Sections 2.1 and 2.3 are extracted for (i) the NCC-selected scans to build the training model of the RF; and (ii) the 3D coregistered test scan to be classified using the built RF model. The above steps are repeated for all of the 60 CT volumes of the 20 subjects.

**Figure 6:**
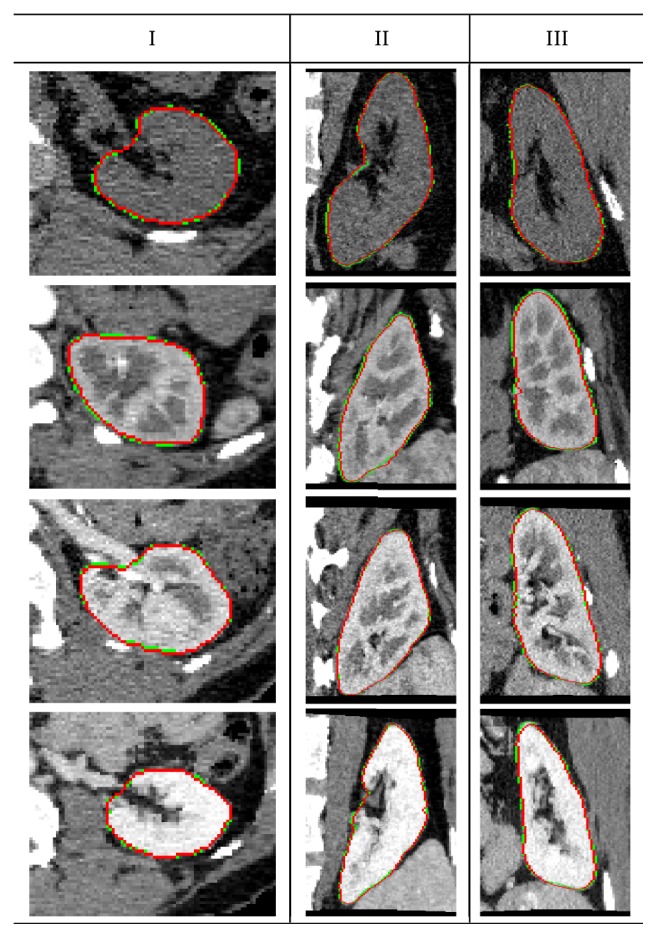
Cross-sectional axial (I), sagittal (II), and coronal (III) segmentation results of our approach for multiple subjects at different contrast-enhancement phases, showing reliable determination of kidney borders (red) compared with the ground truth (green) contours.

Cross-sectional segmentation results in the axial, sagittal, and coronal views using our technique are demonstrated in Figure 6 for CT data from four subjects at different contrast-enhancement phases. The 3D kidney surface is constructed by accounting for the object labels in the output of the RF classifier. Followed by a postprocessing step using a 3D median filter to smooth the noisy output labels of the classifier. The segmentation accuracy of our framework is assessed using the evaluation metrics described in Section 3. The overall accuracy for all subjects in terms of mean and standard deviation is summarized in Table 1.

**Figure 7:**
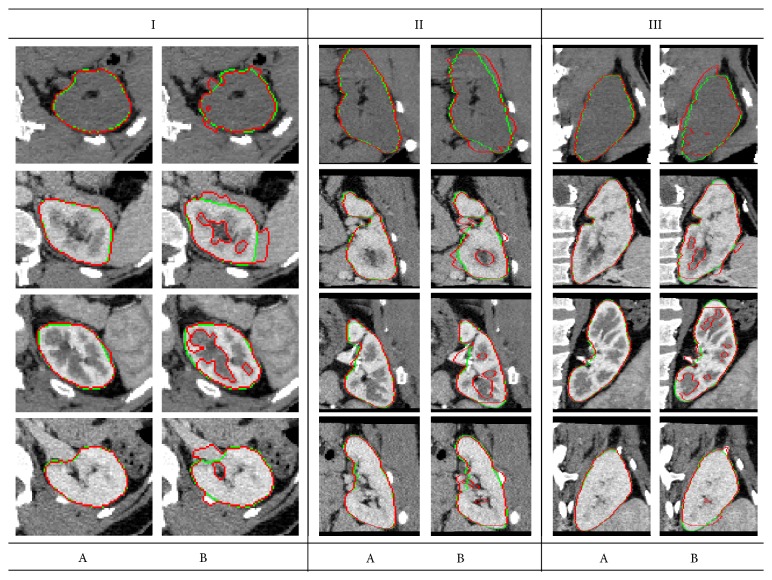
Cross-sectional axial (I), sagittal (II), and coronal (III) segmentation results from multiple subjects at different contrast-enhancement phases of our approach (A) and the approach proposed by Zhang et al. [47] (B). The red and green contours refer to model segmentation and the ground truth, respectively.

In order to demonstrate the high accuracy of our kidney segmentation framework, we compare it with the image segmentation method that was proposed by Zhang et al. [47], which has a freely available software package and thus avoids reimplementing an existing method.  Figure 7 demonstrates sample segmentation results comparing our method versus the approach proposed in [47] on multiple subjects. The results in Figure 7 show reliable determination of the kidney borders of our technique compared to Zhang et al. [47] method. Additionally, a summary of the overall segmentation accuracy of our and Zhang et al. [47] methods, with respect to the ground truth delineation, for all data sets, is given in Table 1. According to the higher DC and lower HD_95_ and PVD values in Table 1, our technique performs notably better compared with [47]. This has been documented using the statistical significance of the statistical paired *t*-test as shown in Table 1 (*p* value is < 0.05).

**Figure 8:**
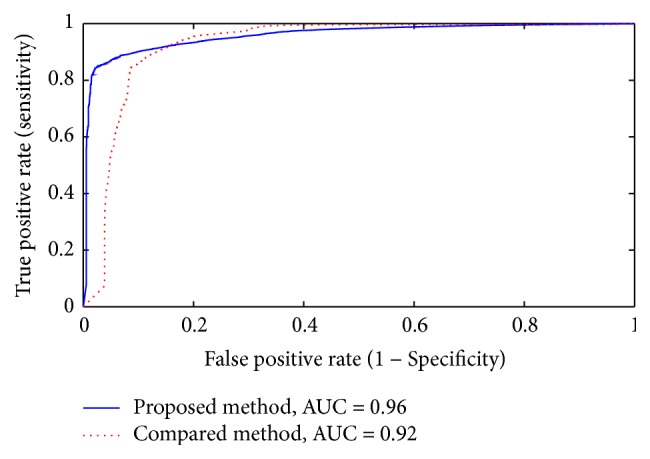
The ROC curves for our segmentation method and the method proposed in [47]. The “AUC” stand for the area under the curve.

In addition to the segmentation evaluation metrics described in Section 2.2, the robustness of our segmentation framework is assessed using the receiver operating characteristics (ROC) [48] as an alternate metric to evaluate the performance of segmentation systems. Generally, the ROC analysis assesses the sensitivity of a segmentation method relative to the choice of its operating point (e.g., a classification threshold). This is achieved by plotting the relationship between the true positive and false positive rates for different operating points.  Figure 8 shows the ROC curves of our method and Zhang et al. [47] approach. The figure clearly demonstrates that our technique attained higher performance compared with [47], as evidenced by the area under the ROC curve (AUC) of 0.96 compared with 0.92 for Zhang et al. approach [47].

## 5. Conclusions

In conclusion, a random forests-based framework is proposed for 3D kidney segmentation from dynamic contrast enhanced abdominal CT images. In order to account for large kidney inhomogeneity and nonlinear intensity variation of the dynamic CT data, our framework integrated two spatial-appearance features, namely, the higher-order spatial interactions features and appearance-based adaptive shape prior features, in addition to the Hounsfield appearance features. Qualitative and quantitative evaluation results confirmed reliable kidney tissue segmentation using our approach at different contrast-enhancement phases of agent transit. This has been evaluated on CT data sets collected from 20 subjects using both volumetric and distance-based evaluation metrics. In the future work we will investigate the addition of other features (e.g., scale space, local binary patterns). Also, we plan to test our framework on larger data sets to assess its accuracy, robustness, and limitation. Ultimately, we plan to include this segmentation approach into a kidney-dedicated CAD system for early detection of acute renal transplant rejection and treatment planning.

## Figures and Tables

**Table 1:** Segmentation accuracy of our method compared with Zhang et al. [47] approach based on the DC, PVD, and BHD_95_ metrics. Note that DC, PVD, BHD_95_, and SD stand for Dice coefficient, percentage volume difference, bidirectional 95th-percentile Hausdorff distance, and standard deviation, respectively.

Metric	Segmentation method		*p* value
	Our	Zhang et al. [47]	
	Mean ± SD	Mean ± SD	
DC (%)	97.27 ± 0.83	91.60 ± 2.29	≤10^−4^
BHD_95_ (mm)	0.93 ± 0.49	5.36 ± 1.12	≤10^−4^
PVD (%)	2.92 ± 2.21	5.00 ± 3.28	≤10^−4^
